# Crystal structure of *fac*-bis­[bis­(pyridin-2-yl)methan­amine]­iron(II) 1,1,3,3-tetra­cyano-2-(di­cyano­methyl­idene)propane-1,3-diide, [Fe(dipa)_2_](tcpd)

**DOI:** 10.1107/S2056989018011143

**Published:** 2018-08-14

**Authors:** Zouaoui Setifi, Peter W. R. Corfield, Fatima Setifi, Bernd Morgenstern, Kaspar Hegetschweiler, Yassine Kaddouri

**Affiliations:** a Département de Technologie, Faculté de Technologie, Université 20 Août 1955-Skikda, BP 26, Route d’El-Hadaiek, Skikda 21000, Algeria; b Laboratoire de Chimie, Ingénierie Moléculaire et Nanostructures (LCIMN), Université Ferhat Abbas Sétif 1, Sétif 19000, Algeria; c Department of Chemistry, Fordham University, 441 East Fordham Road, Bronx, NY 10458, USA; d Fachrichtung Chemie, Universität des Saarlandes, Postfach 151150, D-66041 Saarbrücken, Germany; e Laboratoire de Chimie Appliquée et Environnement (LCAE), Faculté des Sciences, Université Mohamed Premier, BP 524, 60000, Oujda, Morocco

**Keywords:** crystal structure, iron(II) complex, polynitrile anion, terdentate ligands

## Abstract

In the title compound, [Fe(C_11_H_11_N_3_)_2_](C_10_N_6_), the Fe^II^ cation is coordinated by two bis(pyridin-2-yl)methanamine (dipa) ligands and has crystallographic twofold symmetry. There are deviations from ideal octa­hedral geometry due to the steric requirements of the ligands. The polynitrile 1,1,3,3-tetra­cyano-2-(di­cyano­methylid­ene)propane-1,3-diide (tcpd^2−^) dianion is disordered about an inversion center and is not coordinated to the Fe atom. The anion is not planar but has a propeller shape. In the crystal, weak N—H⋯N inter­actions between the amine H atoms of the dipa ligands and two nitrile groups of the anion form an alternating chain of cations and anions related by the *C*-centering of the unit cell.

## Abstract 2

In the title compound, the Fe^II^ cation is octa­hedrally coordinated by two dipa ligands and has crystallographic twofold symmetry. The nonplanar polynitrile tcpd^2−^ anion is disordered about an inversion center.

## Chemical context   

Polynitrile anions have recently received considerable attention in the fields of coordination chemistry and mol­ecular materials (Benmansour *et al.*, 2010 ▸). These organic anions are of inter­est for their ability to act towards metal centers with various coordination modes and for their high degree of electronic delocalization (Miyazaki *et al.*, 2003 ▸; Benmansour *et al.*, 2008 ▸; Yuste *et al.*, 2009 ▸; Atmani *et al.*, 2008 ▸; Karpov *et al.*, 2018 ▸). We are inter­ested in using these anionic ligands in combination with other neutral bridging coligands to explore their structural features and properties relevant to the field of mol­ecular materials exhibiting the spin-crossover (SCO) phenomenon (Setifi *et al.*, 2014 ▸; Dupouy *et al.*, 2009 ▸). In an attempt to prepare such an iron(II) complex using solvothermal synthesis, we obtained instead the title compound *fac*-bis­[bis­(pyridin-2-yl)methanamine]­iron(II) 1,1,3,3-tetra­cyano-2-(di­cyano­methylid­ene)propane-1,3-diide, [Fe(dipa)_2_](tcpd).

## Structural commentary   

**Figure 1:**
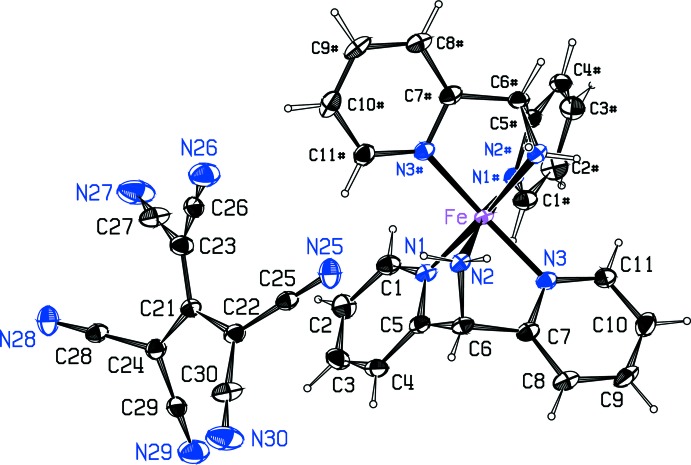
The [Fe(dipa_2_)]^2+^ cation and tcpd^2−^ anion in the title compound. Atoms in the cation with the # suffix are related to the asymmetric unit by (1 − *x*, *y*, 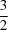 − *z*). Only one orientation of the disordered anion is shown. Displacement ellipsoids are drawn at the probability 50% level.

The structure is built from Fe^II^ cations coordinated by two bis­(pyridin-2-yl)methanamine (C_11_H_11_N_3_; dipa) ligands, and 1,1,3,3-tetra­cyano-2-(di­cyano­methylid­ene)propane-1,3-diide (C_10_N_6_^2−^; tcpd^2−^) anions. The Fe atom lies on a twofold axis, with its coordinated dipa ligands related by the twofold axis (Fig. 1 ▸). The anion lies on an inversion center and is disordered. Detailed geometry of the anion was extracted as described below. The dipa ligand coordinates the Fe atom through the central amino N atom and the two pyridinium N atoms in a *fac* arrangement. The dipa ligand assumes the butterfly conformation found previously (Setifi *et al.*, 2017 ▸), with an approximate mirror plane bis­ecting the ligand, and the pyridine rings are at an angle of 56.66 (6)° to each other. Fe—N distances to the pyridine N atoms average 1.959 (1) Å, slightly shorter than the Fe—N distance of 2.004 (2) Å to the amine group. The five-membered chelate ring angles at the Fe atom are 80.10 (6) and 81.55 (6)°, while the butterfly angle N1—Fe—N3 is 90.10 (6)°. The two independent *trans* angles at Fe in the octa­hedrally coordinated Fe atom are 172.75 (9) and 174.61 (6)°. Otherwise, bond lengths and angles within the ligand are as expected. The tcpd^2−^ anion, which is disordered about a crystallographic inversion center, is propeller-shaped, with approximate *C*_3_ symmetry, and a geometry similar to that described previously by Setifi *et al.* (2015 ▸). The C(CN)_2_ planes are tilted by 31.1 (5), 24.7 (4), and 30.0 (5)° with respect to the C21–C24 central plane. The C—C distances average 1.414 (18) Å, indicative of the *sp* ^2^ hybridization of all the C atoms. The average C—N distance in the CN groups is 1.154 (11) Å.
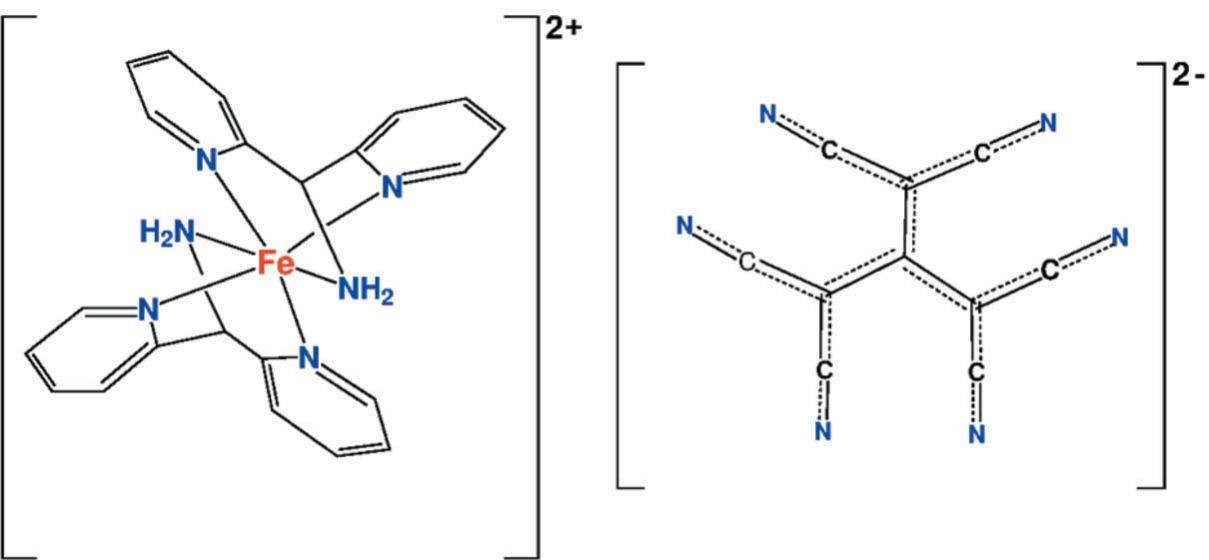


## Supra­molecular features   

**Figure 2:**
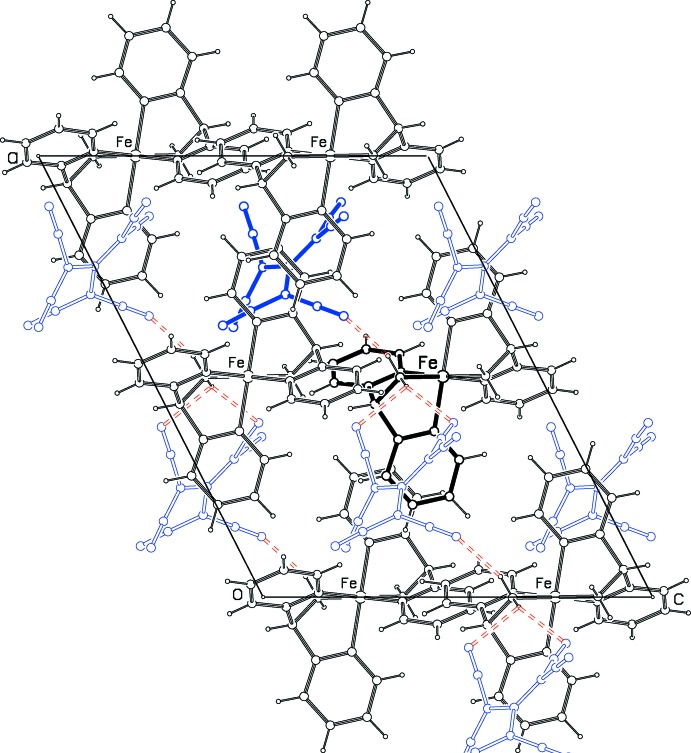
Projection of the structure down the *b* axis, with weak N—H⋯N inter­actions shown as dashed orange bonds. Only one disorder mate for the anion is shown. Cations have more solid bonds. The asymmetric unit is shown in bold.

Fig. 2 ▸ shows the packing of the crystal structure. The cations stack along the *b*-axis direction in columns related by the glide planes and the *C*-centering. The N3/C7–C11 pyridine ring lies almost perpendicular to the *b* axis and partially overlaps the parallel ring related by the center of symmetry at (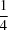, 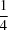, 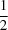). The planes of these pyridine rings are 3.442 (1) Å apart. The anion is disordered about a center of symmetry displaced by *b*/2 from the center of these pyridine rings. Fig. 2 ▸ shows the inter­actions between the amine H atoms and the CN groups. There is an N2—H12⋯N25 hydrogen bond, with N⋯N = 2.95 (1) Å and N—H ⋯ N = 157.1 (2)° (Table 1 ▸). The weaker inter­actions N2—H13⋯N27(*x* − 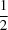, *y* + 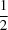, *z*) and N2—H13⋯N28(*x* − 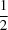, *y* + 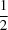, *z*) link the cations and anions into alternating chains along [1
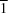
0]. Possible interactions between CN groups and atom H6 are also listed in Table 1 ▸. Other short inter­actions are C6⋯N28(*x* − 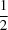, 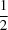 + *y*, *z*) of 2.99 Å and C5⋯N28(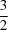 − *x*, 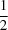 − *y*, 1 − *z*) of 3.11 Å.

## Database survey   

A search for the tcpd^2−^ anion in the Cambridge Structural Database (CSD, Version of 2017; Groom *et al.*, 2016 ▸) produced 49 hits for structures with atomic coordinates available. We selected 20 of these hits for analysis, not using 23 variable-temperature studies and six with disordered tcpd^2−^ anions for which detailed parameters were not available. In nine of the 20 studies, the anion was present in an uncoordinated form, and in the rest, it was coordinated to a first-row transition metal. The bond lengths in the 20 structures analyzed were quite consistent, with sample deviations of 0.013 Å. The two types of C—C distances have the same average shortened distance of 1.417 (1) Å, and the C≡N bond lengths average 1.147 (1) Å, showing the same trends as in the present structure. In all cases, the anion as a whole was nonplanar, with each C(CN)_2_ group twisted in the same direction relative to the central four-atom plane, with an average twist angle of 24.4 (7)°. Presumably, this minimizes repulsion between the N atoms, which carry a partial negative charge. The average twist angle is the same, regardless of whether the anion is coordinated. In an individual structure, the twist angles were invariably scattered, with the average minimum twist angle some 67% of the average maximum twist angle. The twist angles for tcpd^2−^ in the present structure average 28.6 (3)°, higher than the average twist angle in any of the nine free anions reviewed in the CSD.

A search for the dipa ligand yielded nine hits, with one, two, or three dipa mol­ecules coordinated to transition-metal atoms in all cases. There were only three instances of dipa coordinated to an Fe atom, including Setifi *et al.* (2017 ▸).

## Synthesis and crystallization   

The title compound was synthesized solvothermally under autogenous pressure from a mixture of FeSO_4_·7H_2_O (28 mg, 0.1 mmol), dipa (19 mg, 0.1 mmol) and K_2_tcpd (28 mg, 0.1 mmol) in water–ethanol (4:1 *v*/*v*, 20 ml). This mixture was sealed in a Teflon-lined autoclave and held at 423 K for 3 d, and then cooled to room temperature at a rate of 10 K h^−1^ (yield 23%). Red blocks of the title compound suitable for single-crystal X-ray diffraction were selected directly from the synthesized product.

## Refinement   

**Figure 3:**
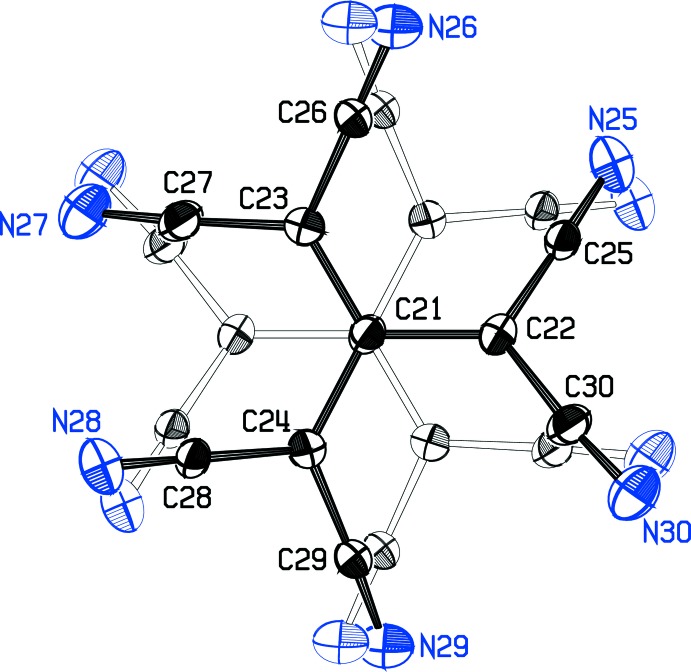
The tcpd^2−^ anion structure superimposed on its disorder mate related by (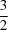 − *x*, 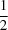 − *y*, 1 − *z*). Atom C21 lies on the center of symmetry at (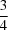, 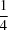, 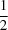).

Crystal data, data collection and structure refinement details are summarized in Table 2 ▸. The tcpd^2−^ anion lies on an inversion center, which requires the anion to be disordered. The three C atoms bonded to the central C21 atom are easily resolved from their centrosymmetric equivalents, but the six CN groups are close enough to a centric array that the disorder mates are not resolved in a difference map. Indeed, a preliminary refinement that constrained the cyanide C and N atoms to centrically related positions converged successfully. This treatment led to unreasonable C—C≡N bond angles, however, and hindered a detailed analysis of the anion geometry. Scrutiny of a model indicated that while the cyanide C atoms might be very close to their centric counterparts, the cyanide N atoms ought to lie far enough apart to refine separately. Accordingly, the positions for the N atoms were calculated manually, assuming linear C—C≡N bonding and typical C—C and C≡N distances. The complete anion could then be refined by tightly restricting differences from threefold symmetry in chemically equivalent C—C distances and C—C—C angles, and refining the C and N atoms first isotropically and then anisotropically. At this point, cyanide C atoms had moved an average of 0.4 Å from their centric images, and cyanide N atoms were at an average distance of 0.5 Å from their images (Fig. 3 ▸). The restraints could now be removed for the final refinements, except that displacement parameters for nitrile groups 25–27 were constrained to be the same as those for nitrile groups 28–30, and a restraint on the anisotropy of C atoms 25–30 was added *via* an ISOR instruction. In a separate refinement, the coordinates of the cyanide C atoms were arbitrarily moved small amounts before the restrained refinements, but the same final structure was obtained. C-bound H atoms were constrained to idealized positions, with C—H distances of 1.00 Å for the CH group and 0.95 Å for aromatic H atoms, and with *U* values set at 1.2 times the *U*_iso_ value of their bonded atoms. The positions of the amino H atoms were refined independently, although their final positions are very close to what would have been predicted. Their *U* values were set at 1.5 times the *U*_iso_ value for N2. We also explored refinements in the space group *Cc*, which would not force disorder on the anion model, nor impose twofold symmetry on the cation. Although the noncentric model refined smoothly, it was abandoned because some of the displacement ellipsoids were unreasonable and convergence occurred at *R*_1_ = 0.043 and *wR* = 0.109, values higher than in our final centric model, even though many more variables were refined in the noncentric model.

## Supplementary Material

Crystal structure: contains datablock(s) I. DOI: 10.1107/S2056989018011143/hb7760sup1.cif

Structure factors: contains datablock(s) I. DOI: 10.1107/S2056989018011143/hb7760Isup2.hkl

CCDC reference: 1860212

Additional supporting information:  crystallographic information; 3D view; checkCIF report

## Figures and Tables

**Table 1:** Hydrogen-bond geometry (Å, °)

*D*—H⋯*A*	*D*—H	H⋯*A*	*D*⋯*A*	*D*—H⋯*A*
N2—H12⋯N25	0.88 (2)	2.12 (3)	2.950 (13)	156.9 (19)
N2—H13⋯N27^i^	0.90 (2)	2.64 (2)	3.459 (8)	151.1 (17)
N2—H13⋯N28^i^	0.90 (2)	2.61 (2)	3.138 (11)	118.2 (16)
C6—H6⋯N25^ii^	1.00	2.47	3.129 (13)	123
C6—H6⋯N28^i^	1.00	2.41	2.986 (13)	116

Symmetry codes: (i) 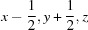; (ii) 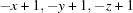.

**Table 2:** Experimental details

Crystal data	
Chemical formula	[Fe(C_11_H_11_N_3_)_2_](C_10_N_6_)
*M*_r_	630.46
Crystal system, space group	Monoclinic, *C*2/*c*
Temperature (K)	162
*a*, *b*, *c* (Å)	17.5394 (7), 13.5094 (7), 13.7913 (6)
β (°)	117.006 (3)
*V* (Å^3^)	2911.5 (2)
*Z*	4
Radiation type	Mo *K*α
μ (mm^−1^)	0.56
Crystal size (mm)	0.39 × 0.20 × 0.02

Data collection	
Diffractometer	Bruker APEXII CCD
Absorption correction	Multi-scan (*SADABS*; Bruker, 2015 ▸)
*T*_min_, *T*_max_	0.667, 0.746
No. of measured, independent and observed [*I* > 2σ(*I*)] reflections	11328, 3227, 2623
*R*_int_	0.035
(sin θ/λ)_max_ (Å^−1^)	0.642

Refinement	
*R*[*F* ^2^ > 2σ(*F* ^2^)], *wR*(*F* ^2^), *S*	0.036, 0.085, 1.04
No. of reflections	3227
No. of parameters	243
No. of restraints	18
H-atom treatment	H atoms treated by a mixture of independent and constrained refinement
Δρ_max_, Δρ_min_ (e Å^−3^)	0.31, −0.42

Computer programs: *APEX2* and *SAINT* (Bruker, 2015 ▸), *SHELXS97* (Sheldrick, 2008 ▸), *SHELXL2017* (Sheldrick, 2015 ▸), *ORTEPIII* (Burnett & Johnson, 1996 ▸) and *publCIF* (Westrip, 2010 ▸).

## supplementary crystallographic information

### Crystal data

**Table:**

[Fe(C_11_H_11_N_3_)_2_](C_10_N_6_)	*F*(000) = 1296
*M**_r_* = 630.46	*D*_x_ = 1.438 Mg m^−^^3^
Monoclinic, *C*2/*c*	Mo *K*α radiation, λ = 0.71073 Å
*a* = 17.5394 (7) Å	Cell parameters from 3760 reflections
*b* = 13.5094 (7) Å	θ = 2.2–27.1°
*c* = 13.7913 (6) Å	µ = 0.56 mm^−^^1^
β = 117.006 (3)°	*T* = 162 K
*V* = 2911.5 (2) Å^3^	Plate, red
*Z* = 4	0.39 × 0.20 × 0.02 mm

### Data collection

**Table:**

Bruker APEXII CCD diffractometer	2623 reflections with *I* > 2σ(*I*)
φ and ω scans	*R*_int_ = 0.035
Absorption correction: multi-scan (SADABS; Bruker, 2015)	θ_max_ = 27.1°, θ_min_ = 2.0°
*T*_min_ = 0.667, *T*_max_ = 0.746	*h* = −22→22
11328 measured reflections	*k* = −17→17
3227 independent reflections	*l* = −17→17

### Refinement

**Table:**

Refinement on *F*^2^	Primary atom site location: structure-invariant direct methods
Least-squares matrix: full	Hydrogen site location: mixed
*R*[*F*^2^ > 2σ(*F*^2^)] = 0.036	H atoms treated by a mixture of independent and constrained refinement
*wR*(*F*^2^) = 0.085	*w* = 1/[σ^2^(*F*_o_^2^) + (0.0355*P*)^2^ + 2.4992*P*] where *P* = (*F*_o_^2^ + 2*F*_c_^2^)/3
*S* = 1.03	(Δ/σ)_max_ < 0.001
3227 reflections	Δρ_max_ = 0.31 e Å^−^^3^
243 parameters	Δρ_min_ = −0.42 e Å^−^^3^
18 restraints	

### Special details

**Table:**

Geometry. All esds (except the esd in the dihedral angle between two l.s. planes) are estimated using the full covariance matrix. The cell esds are taken into account individually in the estimation of esds in distances, angles and torsion angles; correlations between esds in cell parameters are only used when they are defined by crystal symmetry. An approximate (isotropic) treatment of cell esds is used for estimating esds involving l.s. planes.

### Fractional atomic coordinates and isotropic or equivalent isotropic displacement parameters (Å^2^)

**Table:**

	*x*	*y*	*z*	*U*_iso_*/*U*_eq_	Occ. (<1)
Fe	0.500000	0.33107 (3)	0.750000	0.01516 (11)	
N1	0.51003 (9)	0.23776 (11)	0.64840 (12)	0.0171 (3)	
N2	0.49834 (10)	0.42852 (12)	0.63946 (12)	0.0182 (3)	
H12	0.5465 (14)	0.4381 (15)	0.6361 (17)	0.027*	
H13	0.4774 (13)	0.4883 (16)	0.6433 (16)	0.027*	
N3	0.37599 (9)	0.34028 (11)	0.65615 (12)	0.0178 (3)	
C1	0.55086 (12)	0.15040 (13)	0.66787 (15)	0.0220 (4)	
H1	0.573114	0.122972	0.738919	0.026*	
C2	0.56168 (13)	0.09904 (15)	0.58871 (16)	0.0271 (4)	
H2	0.591205	0.037572	0.605156	0.032*	
C3	0.52888 (13)	0.13836 (15)	0.48477 (16)	0.0269 (4)	
H3	0.535829	0.104295	0.429038	0.032*	
C4	0.48589 (11)	0.22779 (14)	0.46310 (15)	0.0223 (4)	
H4	0.462349	0.255803	0.392230	0.027*	
C5	0.47789 (11)	0.27554 (13)	0.54654 (14)	0.0185 (4)	
C6	0.43762 (11)	0.37540 (13)	0.53883 (14)	0.0190 (4)	
H6	0.427589	0.411685	0.470869	0.023*	
C7	0.35729 (11)	0.36624 (13)	0.55295 (15)	0.0194 (4)	
C8	0.27498 (12)	0.38700 (14)	0.47501 (16)	0.0246 (4)	
H8	0.263606	0.403702	0.402782	0.030*	
C9	0.20953 (12)	0.38291 (15)	0.50458 (17)	0.0287 (5)	
H9	0.152425	0.397943	0.453107	0.034*	
C10	0.22812 (12)	0.35672 (15)	0.60976 (17)	0.0288 (5)	
H10	0.183929	0.353757	0.631450	0.035*	
C11	0.31139 (11)	0.33492 (14)	0.68303 (15)	0.0225 (4)	
H11	0.323508	0.315486	0.754795	0.027*	
C21	0.750000	0.250000	0.500000	0.0192 (5)	
C22	0.6860 (2)	0.3267 (3)	0.4533 (3)	0.0222 (8)	0.5
C23	0.8132 (2)	0.2577 (3)	0.6062 (3)	0.0238 (8)	0.5
C24	0.7474 (2)	0.1695 (3)	0.4309 (3)	0.0208 (7)	0.5
C25	0.6592 (6)	0.3810 (9)	0.5196 (6)	0.0197 (11)	0.5
C26	0.8404 (9)	0.3491 (10)	0.6596 (15)	0.0225 (16)	0.5
C27	0.8597 (9)	0.1729 (6)	0.6652 (11)	0.0289 (14)	0.5
C28	0.8232 (6)	0.1203 (9)	0.4490 (6)	0.0197 (11)	0.5
C29	0.6717 (9)	0.1319 (10)	0.3432 (15)	0.0225 (16)	0.5
C30	0.6485 (9)	0.3540 (6)	0.3413 (12)	0.0289 (14)	0.5
N25	0.6372 (6)	0.4221 (10)	0.5748 (6)	0.0298 (14)	0.5
N26	0.8632 (6)	0.4253 (8)	0.7055 (10)	0.0391 (14)	0.5
N27	0.8951 (7)	0.1069 (8)	0.7166 (6)	0.0490 (14)	0.5
N28	0.8855 (6)	0.0826 (10)	0.4618 (6)	0.0298 (14)	0.5
N29	0.6118 (6)	0.0972 (8)	0.2768 (10)	0.0391 (14)	0.5
N30	0.6157 (7)	0.3822 (8)	0.2521 (6)	0.0490 (14)	0.5

### Atomic displacement parameters (Å^2^)

**Table:**

	*U*^11^	*U*^22^	*U*^33^	*U*^12^	*U*^13^	*U*^23^
Fe	0.01275 (17)	0.01604 (19)	0.01247 (18)	0.000	0.00205 (14)	0.000
N1	0.0157 (7)	0.0164 (8)	0.0154 (7)	−0.0002 (6)	0.0038 (6)	0.0006 (6)
N2	0.0149 (7)	0.0163 (8)	0.0201 (8)	0.0001 (6)	0.0051 (6)	0.0007 (6)
N3	0.0170 (7)	0.0163 (8)	0.0173 (7)	−0.0017 (6)	0.0052 (6)	−0.0021 (6)
C1	0.0228 (9)	0.0191 (10)	0.0199 (10)	0.0015 (7)	0.0059 (8)	0.0015 (7)
C2	0.0305 (10)	0.0196 (10)	0.0291 (11)	0.0035 (8)	0.0117 (9)	−0.0027 (8)
C3	0.0300 (10)	0.0281 (11)	0.0233 (10)	−0.0040 (9)	0.0126 (9)	−0.0089 (8)
C4	0.0215 (9)	0.0268 (10)	0.0150 (9)	−0.0046 (8)	0.0051 (8)	−0.0006 (8)
C5	0.0150 (8)	0.0206 (10)	0.0166 (9)	−0.0029 (7)	0.0044 (7)	0.0009 (7)
C6	0.0178 (9)	0.0196 (9)	0.0148 (9)	0.0005 (7)	0.0034 (7)	0.0033 (7)
C7	0.0182 (8)	0.0149 (9)	0.0197 (9)	−0.0008 (7)	0.0040 (7)	−0.0009 (7)
C8	0.0203 (9)	0.0222 (10)	0.0215 (10)	−0.0004 (8)	0.0008 (8)	0.0004 (8)
C9	0.0136 (9)	0.0295 (11)	0.0303 (11)	0.0001 (8)	−0.0013 (8)	−0.0044 (9)
C10	0.0172 (9)	0.0325 (12)	0.0349 (12)	−0.0035 (8)	0.0102 (9)	−0.0087 (9)
C11	0.0194 (9)	0.0246 (10)	0.0215 (9)	−0.0048 (8)	0.0076 (8)	−0.0056 (8)
C21	0.0173 (12)	0.0220 (14)	0.0160 (13)	0.0025 (10)	0.0057 (10)	0.0021 (10)
C22	0.0199 (17)	0.0241 (19)	0.0181 (18)	0.0045 (16)	0.0048 (15)	0.0017 (16)
C23	0.0263 (19)	0.021 (2)	0.0196 (19)	0.0034 (16)	0.0066 (16)	−0.0027 (15)
C24	0.0195 (17)	0.0201 (18)	0.0204 (18)	0.0023 (15)	0.0069 (15)	−0.0019 (15)
C25	0.019 (3)	0.0175 (10)	0.018 (3)	0.002 (2)	0.005 (3)	0.002 (3)
C26	0.022 (3)	0.024 (4)	0.0198 (12)	0.006 (3)	0.008 (2)	−0.003 (3)
C27	0.029 (2)	0.023 (4)	0.0211 (16)	−0.001 (3)	−0.0001 (15)	0.000 (3)
C28	0.019 (3)	0.0175 (10)	0.018 (3)	0.002 (2)	0.005 (3)	0.002 (3)
C29	0.022 (3)	0.024 (4)	0.0198 (12)	0.006 (3)	0.008 (2)	−0.003 (3)
C30	0.029 (2)	0.023 (4)	0.0211 (16)	−0.001 (3)	−0.0001 (15)	0.000 (3)
N25	0.031 (4)	0.0234 (15)	0.041 (5)	0.004 (3)	0.021 (3)	0.002 (4)
N26	0.040 (5)	0.035 (5)	0.032 (4)	−0.001 (3)	0.008 (4)	−0.010 (3)
N27	0.063 (3)	0.045 (2)	0.024 (4)	0.0162 (19)	0.007 (3)	0.009 (3)
N28	0.031 (4)	0.0234 (15)	0.041 (5)	0.004 (3)	0.021 (3)	0.002 (4)
N29	0.040 (5)	0.035 (5)	0.032 (4)	−0.001 (3)	0.008 (4)	−0.010 (3)
N30	0.063 (3)	0.045 (2)	0.024 (4)	0.0162 (19)	0.007 (3)	0.009 (3)

### Geometric parameters (Å, º)

**Table:**

Fe—N1^i^	1.9512 (15)	C7—C8	1.382 (2)
Fe—N1	1.9512 (15)	C8—C9	1.383 (3)
Fe—N3^i^	1.9659 (14)	C8—H8	0.9500
Fe—N3	1.9659 (14)	C9—C10	1.381 (3)
Fe—N2^i^	2.0042 (16)	C9—H9	0.9500
Fe—N2	2.0043 (16)	C10—C11	1.379 (3)
N1—C1	1.343 (2)	C10—H10	0.9500
N1—C5	1.354 (2)	C11—H11	0.9500
N2—C6	1.494 (2)	C21—C23	1.383 (4)
N2—H12	0.88 (2)	C21—C24	1.433 (4)
N2—H13	0.90 (2)	C21—C22	1.447 (4)
N3—C11	1.345 (2)	C22—C25	1.408 (12)
N3—C7	1.354 (2)	C22—C30	1.425 (15)
C1—C2	1.377 (3)	C23—C26	1.406 (14)
C1—H1	0.9500	C23—C27	1.427 (11)
C2—C3	1.385 (3)	C24—C28	1.403 (11)
C2—H2	0.9500	C24—C29	1.424 (15)
C3—C4	1.383 (3)	C25—N25	1.143 (18)
C3—H3	0.9500	C26—N26	1.18 (2)
C4—C5	1.381 (3)	C27—N27	1.133 (15)
C4—H4	0.9500	C28—N28	1.145 (18)
C5—C6	1.504 (3)	C29—N29	1.13 (2)
C6—C7	1.512 (2)	C30—N30	1.161 (16)
C6—H6	1.0000		
			
N1^i^—Fe—N1	99.51 (9)	C4—C5—C6	125.61 (16)
N1^i^—Fe—N3^i^	90.09 (6)	N2—C6—C5	104.56 (14)
N1—Fe—N3^i^	94.59 (6)	N2—C6—C7	103.44 (14)
N1^i^—Fe—N3	94.59 (6)	C5—C6—C7	110.64 (15)
N1—Fe—N3	90.09 (6)	N2—C6—H6	112.5
N3^i^—Fe—N3	172.75 (9)	C5—C6—H6	112.5
N1^i^—Fe—N2^i^	81.55 (6)	C7—C6—H6	112.5
N1—Fe—N2^i^	174.61 (6)	N3—C7—C8	122.63 (17)
N3^i^—Fe—N2^i^	80.10 (6)	N3—C7—C6	111.19 (15)
N3—Fe—N2^i^	95.10 (6)	C8—C7—C6	126.07 (17)
N1^i^—Fe—N2	174.61 (6)	C7—C8—C9	118.46 (18)
N1—Fe—N2	81.55 (6)	C7—C8—H8	120.8
N3^i^—Fe—N2	95.10 (6)	C9—C8—H8	120.8
N3—Fe—N2	80.10 (6)	C10—C9—C8	119.23 (17)
N2^i^—Fe—N2	97.89 (9)	C10—C9—H9	120.4
C1—N1—C5	118.15 (16)	C8—C9—H9	120.4
C1—N1—Fe	129.65 (13)	C11—C10—C9	119.42 (18)
C5—N1—Fe	111.76 (12)	C11—C10—H10	120.3
C6—N2—Fe	98.58 (11)	C9—C10—H10	120.3
C6—N2—H12	108.6 (14)	N3—C11—C10	122.08 (18)
Fe—N2—H12	117.1 (14)	N3—C11—H11	119.0
C6—N2—H13	110.4 (13)	C10—C11—H11	119.0
Fe—N2—H13	114.2 (13)	C23—C21—C24	122.0 (2)
H12—N2—H13	107.5 (19)	C23—C21—C22	120.3 (2)
C11—N3—C7	118.15 (15)	C24—C21—C22	117.6 (2)
C11—N3—Fe	129.34 (13)	C25—C22—C30	116.3 (7)
C7—N3—Fe	112.09 (11)	C25—C22—C21	120.3 (4)
N1—C1—C2	122.49 (17)	C30—C22—C21	123.4 (6)
N1—C1—H1	118.8	C21—C23—C26	122.6 (7)
C2—C1—H1	118.8	C21—C23—C27	121.3 (5)
C1—C2—C3	119.01 (18)	C26—C23—C27	115.9 (8)
C1—C2—H2	120.5	C28—C24—C29	115.2 (7)
C3—C2—H2	120.5	C28—C24—C21	120.0 (4)
C4—C3—C2	119.21 (18)	C29—C24—C21	124.8 (6)
C4—C3—H3	120.4	N25—C25—C22	177.7 (12)
C2—C3—H3	120.4	N26—C26—C23	179.2 (19)
C5—C4—C3	118.67 (17)	N27—C27—C23	176.0 (16)
C5—C4—H4	120.7	N28—C28—C24	177.7 (12)
C3—C4—H4	120.7	N29—C29—C24	176.1 (17)
N1—C5—C4	122.46 (17)	N30—C30—C22	175.0 (12)
N1—C5—C6	111.87 (15)		
			
C5—N1—C1—C2	0.6 (3)	C5—C6—C7—N3	68.77 (19)
Fe—N1—C1—C2	−171.08 (14)	N2—C6—C7—C8	133.42 (18)
N1—C1—C2—C3	−0.4 (3)	C5—C6—C7—C8	−115.1 (2)
C1—C2—C3—C4	−0.3 (3)	N3—C7—C8—C9	1.3 (3)
C2—C3—C4—C5	0.7 (3)	C6—C7—C8—C9	−174.40 (18)
C1—N1—C5—C4	−0.2 (3)	C7—C8—C9—C10	−1.1 (3)
Fe—N1—C5—C4	172.93 (14)	C8—C9—C10—C11	−0.2 (3)
C1—N1—C5—C6	−177.48 (15)	C7—N3—C11—C10	−1.2 (3)
Fe—N1—C5—C6	−4.33 (17)	Fe—N3—C11—C10	170.60 (14)
C3—C4—C5—N1	−0.4 (3)	C9—C10—C11—N3	1.4 (3)
C3—C4—C5—C6	176.44 (16)	C23—C21—C22—C25	−31.1 (7)
Fe—N2—C6—C5	−56.76 (13)	C24—C21—C22—C25	151.7 (6)
Fe—N2—C6—C7	59.12 (13)	C23—C21—C22—C30	147.0 (6)
N1—C5—C6—N2	42.55 (18)	C24—C21—C22—C30	−30.2 (7)
C4—C5—C6—N2	−134.61 (18)	C24—C21—C23—C26	151.9 (9)
N1—C5—C6—C7	−68.22 (19)	C22—C21—C23—C26	−25.2 (10)
C4—C5—C6—C7	114.62 (19)	C24—C21—C23—C27	−22.8 (9)
C11—N3—C7—C8	−0.1 (3)	C22—C21—C23—C27	160.1 (8)
Fe—N3—C7—C8	−173.35 (14)	C23—C21—C24—C28	−26.7 (6)
C11—N3—C7—C6	176.15 (15)	C22—C21—C24—C28	150.5 (6)
Fe—N3—C7—C6	2.94 (18)	C23—C21—C24—C29	152.0 (10)
N2—C6—C7—N3	−42.71 (18)	C22—C21—C24—C29	−30.9 (10)

Symmetry code: (i) −*x*+1, *y*, −*z*+3/2.

### Hydrogen-bond geometry (Å, º)

**Table:**

*D*—H···*A*	*D*—H	H···*A*	*D*···*A*	*D*—H···*A*
N2—H12···N25	0.88 (2)	2.12 (3)	2.950 (13)	156.9 (19)
N2—H13···N27^ii^	0.90 (2)	2.64 (2)	3.459 (8)	151.1 (17)
N2—H13···N28^ii^	0.90 (2)	2.61 (2)	3.138 (11)	118.2 (16)
C6—H6···N25^iii^	1.00	2.47	3.129 (13)	123
C6—H6···N28^ii^	1.00	2.41	2.986 (13)	116

Symmetry codes: (ii) *x*−1/2, *y*+1/2, *z*; (iii) −*x*+1, −*y*+1, −*z*+1.

## References

[bb1] Atmani, C., Setifi, F., Benmansour, S., Triki, S., Marchivie, M., Salaün, J.-Y. & Gómez-García, C. J. (2008). *Inorg. Chem. Commun.* **11**, 921–924.

[bb2] Benmansour, S., Atmani, C., Setifi, F., Triki, S., Marchivie, M. & Gómez-García, C. J. (2010). *Coord. Chem. Rev.* **254**, 1468–1478.

[bb3] Benmansour, S., Setifi, F., Gómez-García, C. J., Triki, S. & Coronado, E. (2008). *J. Mol. Struct*, **890**, 255–262.

[bb4] Bruker (2015). *APEX2*, *SAINT* and *SADABS*. Bruker AXS Inc., Madison, Wisconsin, USA.

[bb5] Burnett, M. N. & Johnson, C. K. (1996). *ORTEPIII*. Report ORNL-6895. Oak Ridge National Laboratory, Tennessee, USA.

[bb6] Dupouy, G., Marchivie, M., Triki, S., Sala-Pala, J., Gómez-García, C. J., Pillet, S., Lecomte, C. & Létard, J.-F. (2009). *Chem. Commun.* pp. 3404–3406. 10.1039/b902339a 19503885

[bb7] Groom, C. R., Bruno, I. J., Lightfoot, M. P. & Ward, S. C. (2016). *Acta Cryst.* B**72**, 171–179. 10.1107/S2052520616003954 PMC4822653 27048719

[bb8] Karpov, S. V., Kayukov, Y. S., Grigorév, A. A. & Tafeenko, V. A. (2018). *Z. Anorg. Allg. Chem.* **644**, 138–141.

[bb9] Miyazaki, A., Okabe, K., Enoki, T., Setifi, F., Golhen, S., Ouahab, L., Toita, T. & Yamada, J. (2003). *Synth. Met.* **137**, 1195–1196.

[bb10] Setifi, Z., Bernès, S., Setifi, F., Kaur, M. & Jasinski, J. P. (2017). *IUCrData*, **2**, x171007.

[bb11] Setifi, F., Milin, E., Charles, C., Thétiot, F., Triki, S. & Gómez-García, C. J. (2014). *Inorg. Chem.* **53**, 97–104. 10.1021/ic401721x 24358979

[bb50] Setifi, Z., Valkonen, A., Fernandes, M. A., Nummelin, S., Boughzala, H., Setifi, F. & Glidewell, C. (2015). *Acta Cryst.* E**71**, 509–515. 10.1107/S2056989015007306 PMC4420140 25995868

[bb12] Sheldrick, G. M. (2008). *Acta Cryst.* A**64**, 112–122. 10.1107/S0108767307043930 18156677

[bb13] Sheldrick, G. M. (2015). *Acta Cryst.* C**71**, 3–8.

[bb14] Westrip, S. P. (2010). *J. Appl. Cryst.* **43**, 920–925.

[bb15] Yuste, C., Bentama, A., Marino, N., Armentano, D., Setifi, F., Triki, S., Lloret, F. & Julve, M. (2009). *Polyhedron*, **28**, 1287–1294.

